# “Willing but unable”: the generative logic and bridging pathways of the digital health divide among rural older adults in China

**DOI:** 10.3389/fpubh.2026.1907383

**Published:** 2026-10-16

**Authors:** Yang Wang, Junli Liang, Zhendong Wu

**Affiliations:** 1 Normal School of Vocational Techniques, Hubei University of Technology, Wuhan, China; 2 School of Law, Humanities and Sociology, Wuhan University of Technology, Wuhan, China

**Keywords:** digital health divide, intergenerational disconnect, rural older adults, spatial justice theory, structural constraints

## Abstract

**Objectives:**

Widespread digital transformation in healthcare has led to serious digital exclusion among rural older adults. This study aims to explore the formation mechanisms of the rural digital health divide in China and propose targeted solutions to address this issue.

**Methods:**

This research adopted reflexive thematic analysis. We conducted in-depth interviews and participant observation with 105 older adults and 20 reference participants (15 adult children/relatives and 5 community workers) recruited from 12 administrative villages in Hubei Province to collect first-hand empirical data.

**Results:**

A typical paradox was identified: rural older adults demonstrate strong willingness and practical needs to use digital health tools, yet remain unable to access the full range of digital health services. The digital health divide is jointly shaped by three interrelated factors: individual physical and cognitive limitations, structural exclusion caused by inadequate digital health infrastructure, and intergenerational disconnection.

**Conclusion:**

Beyond simple individual and infrastructure logic, the rural digital health divide is a complex problem shaped by individual, structural and intergenerational contexts. Informed by spatial justice theory, this study puts forward a systematic optimization framework across physical, social and symbolic spatial dimensions, covering infrastructure upgrading, community capacity building and cultural adaptation. The research findings help eliminate access barriers to digital health services, build an inclusive digital health ecosystem, and promote health equity for rural older populations.

## 1 Introduction

Digital technologies have profoundly reshaped the operation and service delivery of global public health systems, and digital health tools have become pivotal enablers for advancing United Nations Sustainable Development Goal 3 (SDG 3: Good Health and Well-Being) worldwide (1). Online medical registration, mobile payment for medical expenses, remote health consultation, intelligent health monitoring and health information platforms, have become vital means to expand healthcare coverage, optimize medical resource allocation and promote universal health equity (2). However, the digital health divide has become a widespread challenge worldwide (3). Rural older adults, in particular, are severely marginalized and face tremendous difficulties in accessing digital health services, which further widens urban–rural health disparities (4).

Global statistics have reflected the severity of this issue. The *World Social Report 2023* notes that merely 39% of people aged 60 and above worldwide are proficient in using digital devices, and the figure drops sharply among rural older adults in developing countries, greatly restricting their access to modern digital health resources (5). The situation in China is equally concerning. Although the proportion of internet users aged 50 and above has reached 34.1% (6), a striking rural–urban gap still exists. The share of non-internet users in rural China is 54.5%, 20.6 percentage points higher than the national rural average (6). For rural older adults, nearly 30% dare not use smartphones out of fear of operational difficulties, online fraud and information insecurity; over 40% are unable to complete mobile medical payments or operate basic digital health functions independently (7). In essence, rural older adults constitute a typical digitally disadvantaged group, and deficient digital health infrastructure and poor service accessibility remain the core bottlenecks of the digital health divide (8).

The Chinese government has issued a series of policies, such as the *Work Plan for Promoting the High-quality Development of Digital Technology for Aging* and the *Action Plan for Improving the Digital Literacy and Skills of the Whole People*, to eliminate the digital divide for older adults and advance age-friendly digital health development (9, 10). Even so, the digital health divide among rural older adults has not been fundamentally resolved.

Existing studies have investigated the severity, multidimensional causes, adverse impacts and countermeasures of the digital divide among rural older adults from multiple perspectives (11–14). Nevertheless, research gaps remain. Most existing works either focus on macro structural criticism or micro individual ability analysis, while neglecting the interactive relationships between digital health infrastructure, service supply and older adults’ adoption of digital health services (15, 16). Additionally, many policy recommendations are derived from top-down theoretical deduction, rather than based on the real healthcare needs and usage scenarios of rural older adults, leading to low implementation efficiency in practice (17).

Given the above challenges, this study puts forward a core research question: What systematic generative logic, shaped by multiple intertwined factors, underpins the digital health divide among rural older adults? How do inadequate infrastructure and inaccessible health services exacerbate their digital exclusion in the healthcare field? Different from studies that regard rural older adults as passive vulnerable groups, this research adopts an insider perspective and prioritizes their subjective experiences and practical health demands. This paper clarifies the dynamic mechanisms of the rural digital health divide, and explores targeted pathways to build an inclusive digital health environment informed by spatial justice theory.

## 2 Literature review

### 2.1 Concept evolution of digital divide and the emergence of digital health divide

The concept of digital divide was first proposed by the U. S. National Telecommunications and Information Administration (NTIA) in 1999, initially referring to gaps in access to digital devices and network coverage across different social groups (18). With in-depth research, its connotation has been continuously expanded. Norris, Van Dijk and Hacker extended the research scope to gaps in technology usage, operational skills and psychological acceptance. Van Dijk further constructed a multi-stage model of technology adoption (19–22). Gradually, the digital divide has evolved from a single issue of technical access to a multi-dimensional problem involving access, usage, cognition and psychology, deeply embedded in social and cultural contexts.

As digital technologies are deeply integrated into public health and medical services, the digital health divide has developed into an important research branch. It refers to inequalities among different populations, regions and age groups in accessing, using and benefiting from digital health technologies and services. In the Chinese context, this problem is prominently manifested in the overlapping urban–rural divide and intergenerational divide (23–26). Rural areas lag far behind cities in digital health infrastructure construction and public health service layout, and rural older adults bear the dual pressures of physiological aging and backward rural supporting conditions, making the digital health divide increasingly prominent (8).

### 2.2 Causes and impacts of the digital health divide among rural older adults

Local empirical studies in China have analyzed the predicament of rural older adults facing the digital health divide from diverse angles. On the one hand, most digital products and health service platforms lack aging-adaptive design (27). Rural older adults also face shortages of economic and cultural capital (28). Meanwhile, the outflow of young and middle-aged residents leads to the collapse of rural social support networks, all of which combine to place rural older adults at a systematic disadvantage in using digital health services (15). On the other hand, digital technology reverses the traditional knowledge hierarchy in rural society. Younger generations become the “digital authorities” in healthcare scenarios, while older adults lose their traditional cultural dominance, which easily triggers intergenerational tensions and reduces the efficiency of mutual assistance for digital health learning (29, 30).

In terms of practical impacts, limited access to digital health resources and insufficient digital health literacy prevent rural older adults from acquiring medical information, using online healthcare services and enjoying convenient public health support (20, 31–33). This not only weakens their social participation and livelihood security, but also aggravates urban–rural health inequalities, and deprives them of dignity and well-being in later life (34).

Despite abundant research outcomes, limitations still exist. Most current studies only describe the status quo and static causes of the digital health divide (35), lacking systematic analysis on how infrastructure defects and service accessibility problems interact with individual and intergenerational factors. Moreover, existing policy suggestions rarely take the real healthcare demands and usage habits of rural older adults as the starting point, resulting in poor pertinence. This study focuses on infrastructure and service accessibility, and further enriches empirical and theoretical research on the rural digital health divide.

## 3 Analytical lenses and sensitizing concepts

To fill the above research gaps, this study draws on three complementary theoretical lenses as sensitizing concepts (36, 37) to guide data collection and interpretation. Operating at micro, meso, and macro levels respectively, these perspectives inform interview design, support pattern identification during thematic coding, and are iteratively refined in dialog with the empirical data. These lenses are used to support the interpretation of findings rather than to predetermine the thematic structure.

At the micro individual level, the Technology Acceptance Model (TAM) indicates that users’ intention to adopt new technologies is mainly determined by perceived usefulness and perceived ease of use (38–43). For rural older adults, digital health services are closely linked to daily medical treatment, chronic disease management, health monitoring and pension security, so they generally recognize the practical value of such tools. However, physiological decline, psychological anxiety and insufficient operational skills greatly reduce the perceived ease of use. Combined with real healthcare scenarios, this model effectively explains the internal individual barriers to accessing digital health services.

At the meso structural level, Giddens’ Structuration Theory bridges macro structural analysis and micro behavioral observation. The urban–rural dual structure leads to the unbalanced layout of digital health infrastructure and differentiated supply of health services. Defective infrastructure and incomplete service systems restrict rural older adults’ daily use of digital health tools. In turn, the low utilization rate of rural digital health facilities also leads to insufficient follow-up investment, operation and maintenance, forming a mutually reinforcing cycle between structure and individual practice (44).

At the macro spatial value level, Spatial Justice Theory holds that spatial justice includes not only the fair distribution of material resources, but also equal rights for all social groups to exist, participate and express in physical, social and symbolic spaces (45). The unbalanced distribution of digital health resources between urban and rural areas and the inaccessibility of public digital health services are typical manifestations of spatial injustice. The fundamental goal of bridging the digital health divide is to reconstruct an equitable spatial environment. This theory provides clear value orientation for analyzing infrastructure problems and proposing optimization strategies.

## 4 Research methodology and data sources

### 4.1 Research paradigm and stance: a humanistic study based on the standpoint of rural older adults

This study adopts a humanistic qualitative research paradigm. We regard rural older adults as active participants rather than passive recipients of assistance. Digital-health disadvantages are understood not as direct outcomes of defective equipment or platforms, but as outcomes shaped by the interplay of material contexts, individual practical judgments and local social relations. Qualitative methods are used to record their real experiences, practical difficulties, unmet demands, as well as the self-protective coping strategies they develop within restrictive rural settings.

### 4.2 Research sites and participants

#### 4.2.1 Research sites

The field investigation was carried out in Hubei province, a central Chinese province with diverse rural landscapes. Adopting a stratified purposive sampling strategy, we selected 12 administrative villages spanning three typical geographical types (eastern plains, central hills, western mountains) and diverse economic modes (agriculture-dominated areas, labor-exporting areas). This design ensured that the sample captured the key variations in digital health infrastructure conditions and public health service supply across rural settings.

#### 4.2.2 Participants

Core Participants: 105 older adults (age ≥ 55) with long-term residence in the sampled villages, selected through purposive sampling to ensure informational richness and theoretical variation. The sample size was guided by the scope of the research question and the need for geographical and economic diversity, with each participant generating rich, in-depth data. The sample captures diversity in gender, age, education level, marital status, living arrangement, and self-rated health (see Table 1).

**Table 1:** Descriptive statistics of sample characteristics.

Variable	Category	Frequency	Percentage (%)
Gender	Male	47	44.8
	Female	58	55.9
Age	55–59	38	36.2
	60–69	34	32.4
	70–79	26	24.8
	80+	7	6.6
Education level	Primary school or below	60	57.1
	Junior high school	25	23.8
	Senior high school/vocational school/technical school	15	14.3
	College or above	5	4.8
Marital status	Married	72	68.6
	Widowed/Divorced/Unmarried	33	31.4
Living arrangement	Living with children	32	30.5
	Empty-nest (couple only)	49	46.7
	Living alone	24	22.8
Self-rated health	Good	28	26.7
	Fair	52	49.5
	Poor	25	23.8
Total		105	100

All participants have basic contact with mobile phones and other digital devices, and have certain experience of trying digital health functions.

Reference participants: 15 young and middle-aged adults (aged 18–45, children or relatives of the older participants) and 5 community workers. They provided supplementary information about intergenerational assistance and the daily operation of rural public digital health facilities.

Participants were recruited through village committee introductions and door-to-door invitation, with an overall refusal rate below 10% (mainly due to hearing impairment or time constraints). However, selection bias may exist, as participants with closer ties to village authorities may be overrepresented.

### 4.3 Data collection methods: a triangulated strategy of contextualization and participation

#### 4.3.1 In-depth interviews

Semi-structured interviews were conducted with all 105 older adults, with each interview lasting 40 to 60 min. The interview outline centered on digital health usage experience, difficulties caused by equipment and network problems, obstacles to accessing online medical services, and demands for rural public digital health facilities. In addition, semi-structured interviews with 15 young and middle-aged relatives and 5 community workers were used for multi-subject triangulation (detailed in Section 4.5).

All interviews were audio-recorded and transcribed into approximately 280,000 words of textual data. Participants were anonymized with unified coding: P + number (older adults), F + number (young and middle-aged relatives), H + number (community staff).

#### 4.3.2 Situational interviews and participatory tools

We designed the *Digital Story Circles* as a participatory tool, organizing group discussions among 6–8 older adults per session. Discussion themes included using mobile phones for medical consultation, experience of village public smart health terminals, and difficulties in online hospital registration. This method captured shared problems and emotional perceptions regarding digital health access.

#### 4.3.3 Participant observation

Researchers conducted immersive field observations for 2–3 weeks in each sampled village. Key observation contents included the actual usage of digital health devices, operation status of village public digital health service kiosks, network signal stability, and on-site difficulties when older adults used medical-related digital functions. A total of around 50,000 words of field notes and more than 100 scene photos were collected (Figure 1).

**Figure 1:**
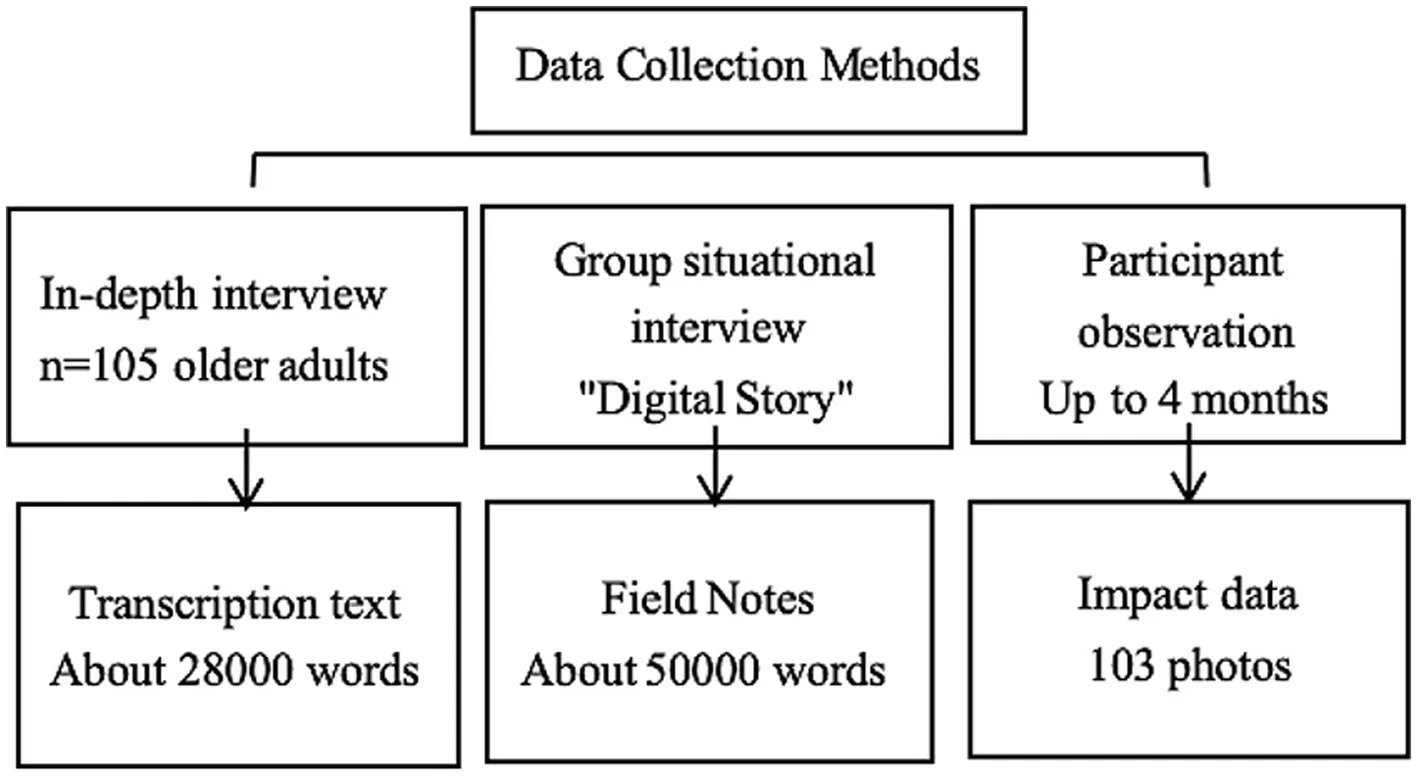
Schematic diagram of data collection.

### 4.4 Data analysis method

This study adopted reflexive thematic analysis for data analysis (46, 47), using NVivo 12 Plus software to code all textual and visual materials. Following data familiarization through repeated reading of transcripts and field notes, the analysis proceeded in a standardized four-stage structure:

Initial coding. Researchers analyzed interview transcripts, field notes and photos line by line. A total of 203 initial codes were extracted, which correspond to the most specific coding items in the tables. These codes cover rural older adults’ attitudes toward digital health learning, daily usage behaviors, diverse demands for digital health services, operational difficulties, equipment and network problems, as well as deficiencies in supporting services and intergenerational support (see Tables 2, 3 for details).

**Table 2:** Demands and current situation of digital device usage among rural older adults.

Third-level coding	Second-level coding	First-level coding	Reference points	Exemplary statements or descriptions
Attitude and willingness toward digital health learning	Positive attitude	Willing to accept	197	*“I am quite interested in using mobile phones to handle medical affairs.”(P14) “Digital health services bring convenience, I am willing to try them.” (P8) “These tools are helpful for seeing a doctor, and I want to learn more.” (P14)*
		Willing to learn	112	
	Negative attitude	Unable and unwilling to learn	36	*“I know these services are useful, but I cannot master the operations and would not use them.” (P49) “The steps are too complicated, I cannot learn it in a short time and give up.” (P65)*
Current situation of digital health usage	Practical daily usage	Making or receiving calls	147	*“I mainly use the phone to make calls and have video chats when consulting doctors.” (P1, P3, P70)*
		WeChat for health-related message check	86	*“I hope to learn to use WeChat to receive health reminders from the village clinic.” (P4)*
		Receiving notification messages	22	*“To check medical insurance notices and health tips on my phone at home.” (P1)*
Digital health service demands	Basic medical service needs	Online hospital registration and appointment	51	*“I want to learning online registration, thus I do not need to go a long way to town hospitals.” (P17)*
		Mobile payment	65	*“I hope to learn how to pay medical bills via mobile phones to save trouble.” (P40)*
		Emergency call or first aid information inquiry	21	*“I want to master emergency calling skills, which may help in critical health situations.” (P20)*
	Health management needs	Online medical insurance inquiry or reimbursement	96	*“I want to check my medical insurance accounts and reimbursement records on my mobile phone.” (P6)*
		Remote health consultation; chronic disease management	19	*“Hope to consult doctors online regularly for my chronic illness.” (P42)“I need online guidance on daily care for chronic diseases.” (P53)*
	Health literacy needs	Learning health or healthcare knowledge	79	*“I want to watch health science videos and learn daily health preservation methods.” (P7);“I hope to acquire more knowledge about disease prevention through mobile phones.” (P35)*
		Audio health lectures	17	*“My eyesight is poor, and I prefer listening to audio health courses.” (P18)*
		Understanding public health policies	13	*“To use my phone to learn about national medical and pension policies.” (P8)*
		Watching science videos on disease prevention	12	*“I want to learn disease prevention knowledge from health science videos.” (P22)*

**Table 3:** Structural determinants of health digital divide among rural older adults.

Third-level coding	Second-level coding	First-level coding	Reference points	Exemplary statements or descriptions
Technology adaptation barriers	Operational and Interaction Difficulties	Operational Challenges	79	*“The operation logic of medical and health apps is too complicated for us to figure out.” (P41); “It is troublesome to complete the procedures for online medical services.” (P5)*
		Mis-touch on Touchscreens	15	*“The touch screen is not sensitive, and I often make mistakes when selecting medical service options.” (P17)*
Interface and cognitive load	Visual/Audio Accessibility Issues		25	*“The font on medical service platforms is too small to read clearly.” (P40) “The text size of hospital appointment pages is not suitable for older people.” (P3)*
	Unfamiliar Icons/Terminology		14	*“Many professional medical terms and function icons on the platform are beyond my understanding.” (P22)*
	Excessive Ads, Poor Information Architecture		48	*“Pop-up advertisements keep appearing when I use medical apps, and I cannot close them easily.” (P23)*
	Rapid Software Updates, Adaptation Difficult		22	*“Health apps update frequently. I just learn one version, and the interface changes again.” (P16)*
Resource and environmental constraints	Inadequate Device and Network Infrastructure	Lack of Devices(Outdated/No Smart Devices)	72	*“Buying a new smartphone and installing network services costs money. Many of us do not have dedicated devices for digital health services.” (P30) “Old mobile phones are too laggy to run medical applications.” (P16)*
		Unstable Network, Poor Signal or High Data Cost	47	*“Network signals in the village are weak; videos for remote health consultation keep buffering.” (P16); “Mobile data fees are high, and I dare not use it to watch health consultation videos.” (P31)*
		Lack of Maintenance & Technical Support	17	*“Once the public health service terminal breaks down, no professional staff will come to repair it in time.” (P32)*
Deficits in education and support systems	Scarcity of Digital Educational Resources	Shortage of Instructors/Volunteers	47	*“There are no professionals in the village to teach us how to use online medical services.” (P15) “We hope someone can come to the village to give lectures on digital health operations.” (P21)*
		Insufficient & Irrelevant Course Materials	56	*“We need simple guidebooks for online registration and medical insurance inquiry, but there are no such materials in the village.” (P43)*
		Lack of Dedicated Learning Spaces	14	*“The village has no fixed place for us to learn how to use digital health facilities.” (P31)*
		Insufficient Funding & Program Sustainability	27	*“Lack of funds and staff makes it impossible to carry out long-term digital health training.” (P34)*
	Fractured Family and Social Support Networks	Ineffective Outreach & Information Dissemination	17	*“We know little about the new digital health policies and public medical service terminals.” (P17)*
		Absence of Instructors (Due to Migration/Lack of Community Support)	77	*“Young people know how to use these medical functions, but most of them work outside the village.” (P14) “No one in the village can give us guidance on digital health operations.” (P69)*
		Lack of Family Support/Patience	13	*“My children are busy and seldom stay at home. They have no time to teach me to use mobile phones for medical treatment.” (P31) “Young people lose patience quickly when teaching me online medical operations.” (P70)*
		Effective Learning with Youth Assistance	35	*“If young people can teach us carefully, we can quickly master the skills of using digital health services.” (P71)*

Generating candidate themes and subthemes. The initial codes were categorized, merged and refined according to semantic and logical relationships to form a series of sub-themes. As the intermediate layer between initial codes and core themes, this step clarified the internal correlations among various influencing factors.

Reviewing and refining themes. All candidate themes were reviewed against the coded data and the entire dataset to ensure internal coherence and distinctiveness. Through iterative refinement, five major themes were finalized: attitudes and status of digital health usage, demands for digital health services, technology adaptation barriers, resource and environmental constraints, and deficits in education and support systems.

Defining and naming themes. Clear conceptual boundaries were established for each final theme, and the logical connections among themes were sorted out to establish the core storyline: The digital health divide among rural older adults is jointly shaped by individual cognition and behaviors, structural constraints (mainly reflected in inadequate digital health infrastructure and poor service accessibility), and intergenerational disconnection.

Figure 2 illustrates the four-stage thematic analysis process, showing how initial codes were progressively clustered into candidate themes and sub-themes, then reviewed, refined, and finalized into five core themes.

**Figure 2:**
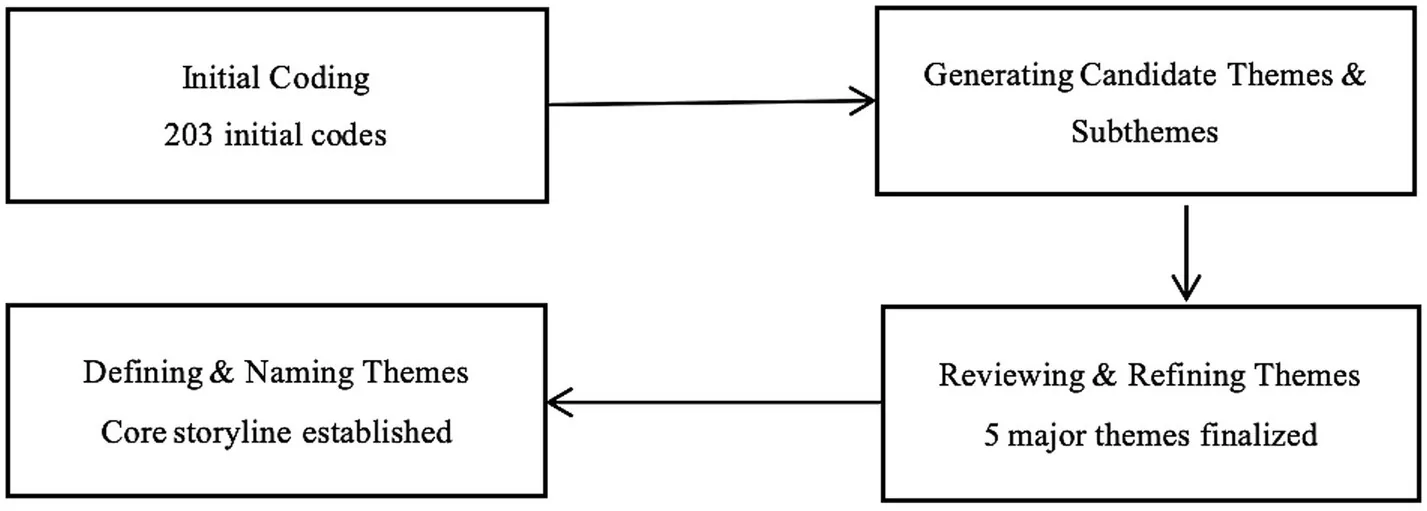
Schematic diagram of four-stage thematic analysis process.

The analysis was primarily data-driven, while the theoretical lenses introduced in Section 3 (TAM, Structuration Theory, and Spatial Justice Theory) were used to support interpretation and theme naming rather than to predetermine the thematic structure. Coding was conducted by the primary researcher and reviewed by a second qualitative researcher; disagreements were resolved through peer debriefing.

### 4.5 Research reliability, validity, and ethics

Data triangulation combining interviews, field observation and group discussions was adopted to ensure research reliability and validity. Specifically, data from 15 young/middle-aged relatives and 5 community workers were systematically triangulated against the older adult sample: relatives’ accounts corroborated the intergenerational disconnect findings, while community workers’ perspectives validated the structural constraints related to public facility maintenance and service delivery.

The research team comprised researchers with backgrounds in sociology, public health, and gerontology, all trained in qualitative methods and age-appropriate communication prior to fieldwork. Reflexive journals were maintained throughout data collection and analysis to document how researchers’ academic backgrounds and participant interactions may have influenced data interpretation.

All participants signed informed consent before the research. Audio recording and photographing were carried out only after obtaining explicit permission. All personal information and place names were fully anonymized. During fieldwork, researchers abided by the *do no harm* principle and provided voluntary guidance when older adults encountered difficulties in using digital health devices.

## 5 Research findings

The research reveals that rural older adults are not passively excluded from the digital health system. On the contrary, they have strong practical demands for online medical registration, remote health consultation, medical insurance inquiry, mobile medical payment and other digital health services. However, their actual engagement remains constrained less by a lack of willingness than by imperfect digital health infrastructure and poor service accessibility, and their hesitations often reflect active risk assessment and self-protective strategies rather than mere inability. Their digital health usage status is far more complex than the simple label of “unable to use digital products”, older adults navigate structural constraints with strategic caution, and infrastructure constraints run through every link of their access to digital health services.

### 5.1 The realities of the digital health divide: the practical paradox of high willingness vs. narrow application

*“My daughter installed WeChat and Alipay on my phone. She told me these apps are necessary for seeing a doctor and checking medical insurance now. I really want to make online appointments and receive medical reimbursements, which can save me a long trip to the town hospital. But I only dare to answer calls and listen to voice messages...... I am afraid of popping advertisements and pressing wrong buttons......I want to learn in my heart, but I dare not operate.*” (P23).

This statement from a 68-year-old rural older woman summarizes the core paradox of the digital health divide: rural older adults have high willingness and strong demand for digital health services, while their operational skills are insufficient and the scope of actual usage is extremely limited. Such hesitation reflects more than insufficient operational capacity, it also embodies older adults’ deliberate risk evaluation and self-protective responses amid unfriendly digital environments. This phenomenon reflects the structural mismatch between digital health service supply, infrastructure layout and the real healthcare needs of rural older adults.

In terms of learning attitude and demand, most rural older adults hold a positive attitude toward digital health tools. Statistical results show that the frequency of participants expressing “*willing to accept and learn digital health functions*” reaches 309, while only 36 cases indicate “*unable and unwilling to learn*” (see Table 2). Their learning motivation can be divided into active learning and passive learning: the former is driven by emotional and practical healthcare needs, such as consulting doctors via video calls and learning health preservation knowledge; the latter comes from rigid requirements in real medical scenarios, such as mandatory QR code scanning for medical treatment and online verification for medical insurance. A small number of older adults refuse to learn, mainly due to expensive smart devices, poor network conditions or long-term reliance on traditional offline medical services.

The digital health demands of rural older adults are diverse and closely combined with daily life, including medical treatment registration, medical insurance inquiry, chronic disease management, health knowledge learning and remote consultation. However, limited by backward infrastructure and unreasonable service design, their actual digital health behaviors are highly restricted. Most older adults can only complete basic call and message receiving operations, and very few can independently use core digital health functions. Digital health products and platforms designed for urban users fail to adapt to rural scenarios, and defective infrastructure directly cuts off their access to digital health resources, forming the dilemma of *having devices but no usable health functions, having network but no accessible health services*.

### 5.2 The shaping mechanisms of the digital health divide: individual, structural, and generational factors

The digital health divide among rural older adults is the result of the joint action of three major factors: individual constraints, structural exclusion dominated by digital health infrastructure and service defects, and intergenerational disconnection.

#### 5.2.1 Individual constraints: the digital health learning paradox of “willing but unable”

From the individual perspective, various operational and interactive barriers form the “willing but unable” paradox among rural older adults when using digital health services (see Table 3). The complicated operating logic of medical and health applications and cumbersome online service procedures bring prominent operational challenges (Reference Points = 79). Meanwhile, insensitive touch screens frequently lead to misoperation when selecting medical service options (Reference Points = 15). In terms of interface experience, medical platforms are designed with overly small fonts and poor audio-visual adaptation for older adults (Reference Points = 25). A large number of unfamiliar icons and professional medical terminology also increase understanding difficulties (Reference Points = 14). In addition, pervasive pop-up advertisements and disordered page layouts interfere with normal use (Reference Points = 48). Frequent version updates of health applications and continuous interface changes further raise the learning and adaptation threshold (Reference Points = 22). Such practical usage difficulties are not merely technical inconveniences, they reflect a fundamental mismatch between the design logic of digital health applications and the practical needs of rural older adults. Most digital-health tools are primarily developed with the usage contexts of mainstream urban users in mind, while the heterogeneous physiological conditions, cognitive habits and daily scenarios of rural older adults have not been sufficiently incorporated into product iteration. Observed barriers including intrusive pop-up advertisements, undersized display fonts, and frequent interface updates, therefore, are not isolated technical bugs. Instead, they represent systematic design gaps that place the burden of adaptation onto older end-users.

These mismatches set restrictive contextual conditions, yet, technical limitations alone cannot fully explain exclusion. Faced with cumbersome interfaces and potential operational risks, rural older adults often limit their own usage scope or avoid unfamiliar functions. Together with age-related physiological and cognitive shifts, these contextual constraints and individual adaptive responses give rise to individual-level barriers to digital-health participation in rural China.

#### 5.2.2 Structural constraints: triple capital deprivation and the systemic generation of digital health divide

Field investigation finds that many rural public smart health service kiosks are left idle. “*This equipment is just for show. The network is often disconnected and responds slowly. No one teaches us how to use it. In the end, we still have to take a bus to the town to handle medical affairs.”* (P32).

This kind of idle public facility is a typical case of disembedded infrastructure: the hardware is in place, but supporting network, operation, maintenance and guidance services are missing, resulting in complete loss of health service functions. Community workers corroborated this finding, noting that public digital health terminals were often installed as top-down policy mandates without sustained funding for maintenance or dedicated staff for operation guidance. As one community worker explained, the equipment “*breaks down and no one comes to fix it, because the township health center has no budget for maintenance*” (H3). This perspective confirms that the “disembedded infrastructure” problem is not merely a perception of older adults, but a structural reality acknowledged by frontline service providers.

Such idleness reflects deeper institutional characteristics of rural public-service governance rather than mere equipment defects. Digital-health terminals are often rolled out as top-down policy initiatives, where hardware quantity serves as a measurable performance indicator, while ongoing expenses for network operation, equipment repair and grassroots guidance lack matching fiscal allocations. Misalignment between short-term policy assessment cycles and long-term service-maintenance requirements further reinforces this “build-and-neglect” pattern, turning hardware deployment into a symbolic policy outcome rather than a practical instrument for residents’ well-being.

Combined with coding data, structural constraints rooted in triple capital deprivation fall into three categories (see Table 3). First, economic capital shortage is reflected in inadequate hardware and network infrastructure: many rural families lack dedicated smart devices, outdated phones cannot run medical applications stably (Reference Points = 72), unstable networks cause buffering during remote consultation (Reference Points = 47), and public terminals cannot receive timely maintenance (Reference Points = 17). Second, cultural capital deficiency is embodied in unreasonable platform design: complex operations, inappropriate interfaces, obscure terminology and frequent updates, which do not match older adults’ usage habits and cognitive characteristics. Third, social capital loss appears in the absence of supporting education and social support networks: rural areas lack professional instructors and volunteers (Reference Points = 47), practical guidance materials and fixed learning venues are insufficient (Reference Points = 56, 14), limited funds make long-term training unsustainable (Reference Points = 27), and policy publicity is ineffective (Reference Points = 17). The combined effect of these three capital deficits continuously forms and solidifies the digital health divide.

#### 5.2.3 Generational disconnect: power reversal and emotional suppression

Within the diverse social contexts of rural China, digital health services can reshape the traditional intergenerational relationship in certain family and community settings, creating generational disconnect featured by power reversal and emotional suppression (see Table 3). These dynamics are not uniform across all rural households, rather, they are shaped by factors such as family economic conditions, the availability of local care arrangements, and the strength of village social networks. In traditional rural society, older adults typically hold a dominant position by virtue of life experience, while young people become the main masters of digital operation skills in the digital health era, resulting in obvious intergenerational power reversal.

A large number of young and middle-aged laborers migrate to cities for work, leaving no on-site instructors in rural communities (Reference Points = 77). Even if older adults turn to their family members for help, busy work and poor patience make family guidance ineffective (Reference Points = 13). Interviews with adult children corroborated this intergenerational tension. Many acknowledged that while they recognized the importance of helping their parents use digital health services, the pressures of migrant work and urban living left limited time and patience for remote teaching. As one adult child noted, “*I tell my parents to just wait until I come back for the holidays, because teaching them over the phone is too frustrating*”. This perspective confirms that the intergenerational disconnect is not solely attributable to older adults’ learning difficulties, but is co-produced by the structural constraints of rural labor migration and the emotional dynamics of intergenerational communication.

Such ineffective intergenerational reverse mentoring cannot be simply reduced to individual laziness or impatience. Essentially, it stems from the structural transformation of intergenerational knowledge authority triggered by digitalization: young migrant groups possess dominant digital information capital, while older adults become dependent learners, reversing the traditional experience-based hierarchy. Coupled with long-term urban–rural spatial separation and divergent life rhythms, this power shift expands cognitive and emotional distance between generations, ultimately weakening the effectiveness of family digital support.

Under such circumstances, many rural older adults choose to suppress their learning demands to avoid troubling others. The extent of such emotional suppression, however, may vary across families with different socioeconomic conditions and intergenerational relationship qualities. On the contrary, older adults can quickly master relevant operational skills if young people provide patient guidance (Reference Points = 35). The dual predicament of physical absence and emotional suppression makes the intergenerational reverse mentoring mechanism ineffective, which further widens the digital health divide.

## 6 Bridging pathways: reconstructing digital solidarity informed by spatial justice

Building on the thematic findings above, which identify individual constraints, structural exclusion, and intergenerational disconnection as three interrelated layers of the digital health divide, this study draws on spatial justice theory as an integrative framework to organize targeted solutions. The structural exclusion identified in the thematic analysis corresponds to the physical spatial dimension (infrastructure and service accessibility), intergenerational disconnection and the absence of social support networks map onto the social spatial dimension, and individual cognitive barriers coupled with culturally insensitive design reflect challenges in the symbolic spatial dimension. Thus, the digital health marginalization of rural older adults is essentially a typical spatial injustice: they are trapped by backward infrastructure in physical space, lack learning and assistance resources in social space, and their healthcare needs are ignored in the design of digital health products and services. Based on spatial justice theory and targeting infrastructure upgrading and health service accessibility improvement, this study proposes systematic solutions from physical, social and symbolic spatial dimensions to break the bottlenecks of the digital health divide (See Table 4).

**Table 4:** Bridging pathways for the digital health divide.

Spatial dimension	Current challenges	Bridging pathways	Concrete measures and examples
Physical space	Unstable network signals, insufficient intelligent devices, idle public digital health facilities and disconnected offline-health services	Optimize digital health infrastructure & implement targeted subsidies	*Establish township-level Digital Integration Service Centers dedicated to health facilities and network maintenance (F13); Provide subsidies for basic smart terminals and low-cost data packages for low-income, older adults to access digital health services (H3); Deploy public digital health service terminals at rural clinics, marketplaces and grocery stores where older adults frequently gather (H4)*
Social space	Shortage of digital health training resources, inadequate on-site guidance and fractured intergenerational assistance mechanisms	Community empowerment & intergenerational co-learning for digital health	*Promote the “Time Bank + Community Digital Health Leader” model to organize regular skill training (H3); Develop family digital co-learning platforms for remote guidance on medical service operations (F3); Compile localized practical courses, including online medical registration guidance, mobile medical payment tutorials and medical insurance inquiry guides (F13)*
Symbolic space	Older adults’ voices and health needs are ignored; lack of an inclusive atmosphere for digital health adoption	Revitalize rural local culture & foster an age-friendly digital health culture	*Launch projects to record traditional health preservation knowledge and rural medical customs via digital devices (H1); Implement village oral history and health experience recording initiatives to enhance older adults’ sense of value (H2); Advocate a tolerant atmosphere for “slow learning” and push for age-adaptive redesign of digital health platforms(H3)*

### 6.1 Physical spatial justice: from “disembedded infrastructure” to grounded digital health services

The primary task is to realize the equitable distribution of digital health infrastructure between urban and rural areas and solve the problems of idle facilities and poor network quality.

First, optimize the digital health infrastructure network. Change the traditional logic of prioritizing construction over operation. Set up full-time digital health service facilitators at the township level, responsible for equipment maintenance, network debugging and on-site skill guidance. Arrange public digital health terminals in rural clinics, grocery stores and marketplaces where older adults frequently gather, to integrate digital health services into their daily life.

Second, implement targeted subsidy policies for vulnerable older groups. Provide subsidies for basic smart terminals and low-cost data packages for low-income, advanced-age and empty-nest rural older adults, reduce their economic burden of using digital health tools, and guarantee their basic rights to access public health services in the digital era.

### 6.2 Social spatial justice: from “educational desertification” to “community-based empowerment”

On the basis of improving hardware infrastructure, we need to build a complete supporting service system to solve the problem of “*having equipment but no one to guid*e”.

First, build a community-based and intergenerational co-learning support system. Promote the model of community digital health leaders, encourage local skilled older adults and village volunteers to provide regular digital health skill training, and realize sustainable rural mutual aid (44). Advocate the new concept of “digital filial piety”, encourage migrant children to provide patient remote guidance on digital health operations, and transform simple video communication into targeted health assistance.

Second, develop localized practical courses adapted to rural healthcare demands. Combined with real scenarios such as offline medical treatment, medical insurance inquiry and chronic disease management, compile illustrated guides for online registration, mobile medical payment and remote consultation, and launch dialect-based video tutorials. Make skill training closely connected with the actual health needs of rural older adults (48, 49).

### 6.3 Symbolic spatial justice: cultivate an inclusive digital health culture

Promote the aging-adaptive transformation of digital health products, abandon the single efficiency-oriented design concept, and fully consider the physiological characteristics and usage habits of rural older adults. Technical optimisation, however, cannot solve digital-health inequities on its own. Reduce redundant pop-up advertisements, enlarge fonts and optimize touch screen sensitivity. At the social level, foster an inclusive cultural atmosphere that tolerates “slow learning,” eliminate the stereotype that older adults reject new technologies, and guide the whole society to pay attention to the digital health needs of rural aging populations (50).

Fostering an inclusive digital culture requires moving beyond the ethos of efficiency supremacy to embrace “slow learning.” From a cultural psychology perspective, attitude formation is influenced by socio-cultural environment, individual experience, and group influence. This is particularly salient in rural China, where traditional values such as strong place attachment and contentment with one’s lot may predispose older adults to conservative attitudes toward new technologies (51). Community-level initiatives can counter this by: utilizing rural broadcasts to share peer success stories (49), developing engaging, interest-based materials as suggested by respondents, “use more pictures and animation videos” (F13), and organizing intergenerational practice activities with targeted youth assistance, “bring youth into rural areas for support”(H2). These strategies help older adults personally experience the tangible benefits of digital technology, transforming abstract anxiety into embodied confidence.

## 7 Conclusion and discussion

### 7.1 Conclusion

This study focuses on the digital health divide, taking rural older adults in China as the research sample. Empirical results prove that the digital health exclusion of rural older adults is not a simple individual technical problem, but a comprehensive social issue formed by the interaction of individual constraints, structural exclusion and intergenerational disconnection. The three factors form a vicious *constraint-exclusion-disconnect* cycle, which continuously solidifies the rural digital health divide. Informed by spatial justice theory, this study constructs a three-dimensional governance system covering infrastructure upgrading, social service support and inclusive cultural construction. The proposed pathways focus on delivering grounded digital health services, empowering rural communities and reshaping inclusive culture, which can effectively break infrastructure bottlenecks and improve the accessibility of digital health services for rural older adults. This research enriches empirical studies on the digital health divide in public health, and provides practical references for optimizing rural digital health infrastructure layout and promoting healthcare equity.

### 7.2 Discussion

This study reveals the generative mechanisms of the rural digital health divide through qualitative investigation. The research findings engage in dialog with prior literature and further extend relevant theories in the field of digital health and aging studies.

First, this study advances Van Dijk’s model by identifying a generational dimension that shapes and solidifies the rural digital health divide in China. As illustrated in Section 5.2.3, this dimension is marked by intergenerational power reversal, emotional self-silencing and cognitive dissonance (21). Digital health technologies have begun to challenge, and in certain contexts may partially overturn, the traditional age-based power structure in rural areas. In line with Hofstede’s cultural dimensions theory, the traditional high power distance culture has been subverted: young people dominate digital operation skills while older adults lose their conventional cultural authority, which intensifies intergenerational barriers (52, 53).

The concept of digital reverse mentoring is also enriched in this research. For rural migrant families, remote guidance via WeChat combines skill teaching with emotional companionship. However, due to rural labor outflow and broken family support systems, this mentoring model often fails. Many older adults restrain their learning needs to avoid troubling their children (P24), while stereotypes about older people also reduce young people’s willingness to offer help. Therefore, these findings suggest that rebuilding intergenerational solidarity may require community support and targeted social policies, though the specific effectiveness of such interventions warrants further empirical testing. This study provides a localized theoretical perspective for analyzing intergenerational interactions within the digital health divide.

Second, this study extends the application scope of the Technology Acceptance Model (TAM). For rural older adults in China, the notable gap between perceived usefulness and perceived ease of use in adopting digital health services is not merely an individual cognitive problem, but is fundamentally shaped by multi-layered structural barriers. Rural older adults hold strong learning intentions mainly because digital health tools can help them access medical services and maintain family connections, which reflects high perceived usefulness (54). However, their perceived ease of use is greatly undermined by age-related physiological decline, prevalent technological anxiety, and the lack of age-adaptive design on mainstream medical platforms, such as tiny fonts and intrusive pop-up advertisements. This conclusion echoes the research of Wang et al. on the psychological digital divide (40). More importantly, this study verifies that structural constraints including inadequate digital health infrastructure and insufficient skill training can continuously reshape users’ perceptions of digital health technologies. Accordingly, TAM-related analyses should not be confined to the individual cognitive level, but need to incorporate critical reflections on social and structural environments.

Third, based on capital theory, this study confirms that rural older adults suffer from mutually reinforcing deprivation of economic capital, cultural capital and social capital. Economic capital shortages are mainly reflected in insufficient smart devices and unaffordable mobile data; cultural capital deficiency manifests as the lack of digital health literacy and basic educational foundation; fractured intergenerational support represents the loss of social capital. This viewpoint is consistent with the argument proposed by Shen Guanghui et al. that triple capital deprivation aggravates digital exclusion (15). The three types of capital deficits do not exist independently, but form a vicious cycle: Insufficient economic capital prevents older adults from obtaining basic digital health equipment and network services, which hinders the accumulation of digital health-related cultural capital through practical use. The loss of social capital further magnifies difficulties in operating digital health tools, turning individual skill deficiencies into comprehensive systemic exclusion. This research deepens the understanding of triple capital deprivation, and clarifies that the rural digital health divide is essentially a product of structural spatial exclusion. The unfair allocation of digital health resources across urban and rural areas likely further consolidates the disadvantaged status of rural aging populations, a causal pathway that future quantitative studies could formally test. However, structural constraints do not fully determine outcomes. Capital deprivation and infrastructure gaps set important boundaries for digital-health practice, yet they do not generate inequality in isolation. Rural older adults’ own risk perceptions, adaptive behaviors and interpersonal interactions constantly mediate how technical resources translate into real-world health benefits.

Fourth, practically, the research results offer references for improving rural digital health infrastructure, optimizing community training mechanisms and fostering an age-friendly digital culture. Informed by the principle of spatial justice, targeted policy arrangements and community support can effectively break the multiple constraints faced by rural older adults in using digital health services.

### 7.3 Limitations

Several limitations of this study should be noted. First, all participants were recruited from 12 administrative villages in Hubei Province. While this sample captures central China’s agrarian context, rural areas in eastern coastal and western regions may differ in digital health infrastructure, economic conditions, and cultural norms. Thus, the transferability of our findings beyond central China warrants caution, and future multi-region studies are needed. Second, the exclusive use of qualitative methods prevents us from quantifying the relative contribution of individual influencing factors. Mixed-methods designs that combine qualitative case studies with large-scale surveys are encouraged to address this gap. Third, the identified mechanisms of intergenerational power reversal and emotional suppression may manifest differently across families and communities with varying socioeconomic conditions and intergenerational relationship qualities, and should thus be interpreted as context-specific tendencies rather than universal patterns. Finally, this cross-sectional study cannot reflect long-term evolutions of digital health facilities and older adults’ adoption behaviors. Longitudinal tracking is warranted for further research.

## Data Availability

The raw data supporting the conclusions of this article will be made available by the authors, without undue reservation.
